# Bridging the gap between research-based knowledge and clinical practice: a qualitative examination of patients and physiotherapists’ views on the Otago exercise Programme

**DOI:** 10.1186/s12877-019-1309-6

**Published:** 2019-10-21

**Authors:** Hilde Worum, Daniela Lillekroken, Birgitte Ahlsen, Kirsti Skavberg Roaldsen, Astrid Bergland

**Affiliations:** 1 Department of Physiotherapy, Faculty of Health Sciences, Oslo Metropolitan University, Oslo, Norway; 2 Department of Nursing and Health Promotion, Faculty of Health Sciences, Oslo Metropolitan University, Oslo, Norway; 3 Department of Neurobiology, Health Sciences and Society, Karolinska Institutet, Stockholm, Sweden; 4 Department of Research, Sunnaas Rehabilitation Hospital, Oslo, Norway

**Keywords:** Experience, User views, Fall prevention, Otago exercise Programme, Research-based knowledge, Clinical practice, Physiotherapy, Older adults

## Abstract

**Background:**

Falls and fall-related injuries exacerbate the health problems of older adults, and they are a public health concern. Despite an abundance of research, the implementation of evidence-based fall prevention programs has been slow and limited, additionally and these programs have not reduced the incidence of falling. Therefore, the primary objective of the present study was to examine patients and physiotherapists’ views on the factors that influence the implementation of the community- and evidence-based Otago Exercise Programme for fall prevention.

**Methods:**

We conducted eight in-depth interviews with physiotherapists and patients, and a focus group interview with 12 physiotherapists and authority figures who represented local hospitals and municipalities. The resultant qualitative data were subjected to thematic analysis.

**Results:**

The analysis yielded two main themes: *the researcher’s role and position in the implementation process* and *the tension between research-based knowledge and clinical practice*. The participants believed that research-based knowledge can address the challenges of clinical practice. Further, the patients reported that the fall prevention program made them feel safe and enhanced their ability to cope with daily life. The physiotherapists also observed that research findings do not readily translate into clinical practice. Further, they contended that research-based knowledge is not universal and that it cannot be generalized across different contexts; instead, it must be adapted and translated into a user-friendly language. The findings suggest that the application of research-based knowledge does equate to *filling up empty jars* and that *research-based knowledge does not flow from the expert to the non-expert as water through a tube.* Indeed, physiotherapists and patients are not *tabula rasa.* Additionally, the participants believed that researchers and stakeholders must think critically about who has the power and voice to create *a common understanding.*

**Conclusions:**

Our findings delineate the means by which the gap between research and practice regarding the Otago fall prevention program can bridged. The program can guide clinical work and provide important information that can be used to improve the quality of other fall prevention programs. However, the research-based knowledge that it confers must be adapted for use in clinical contexts.

## Background

There is a global emphasis on the need for clinical practice, education, and research [1–6] that is based on the best available research evidence [7]. Evidence-based practice (EBP) refers to the translation and integration of the best available research-based knowledge, clinical expertise, and patient characteristics and preferences [8]. In this manner, people can receive the most effective evidence-based healthcare [1–6]. Knowledge translation is the process by which knowledge travels from its place of origin, namely, the research environment, to the context in which it can influence clinical practice and patient care [9, 10]. Straus et al. [11] emphasized the distinction between knowledge translation and research translation. Specifically, whereas the latter refers exclusively to the communication and use of research findings, the former encompasses all means of knowledge acquisition. The term *knowledge* encompasses many types of evidence such as research data, local (administrative) data, evaluative findings, organizational priorities, organizational culture and context, patient experiences and preferences, and resource availability.

Falls and fall-related injuries (e.g., hip fractures) exacerbate the health problems of older adults, and they are a public health concern [12–15].. The World Health Organization [16] defines a fall as *an unexpected event where the participant comes to rest on the ground, floor, or lower level.* During the last 20 years, researchers have reported that approximately one-third of community dwellers who are above the age of 65 years experience a fall annually [12–14, 17]. Therefore, the prevention of falls among older adults has emerged as an international health priority [4, 18, 19]. The conclusions of systematic reviews and meta-analytic studies support the effectiveness of fall prevention programs [14, 15, 19–31]. Previous research has shown that the most powerful means to prevent falls is exercise interventions [27]. Physiotherapists often provide such types of intervention; therefore, they play a significant role in fall prevention [32].

The evidence-based Otago Exercise Programme (OEP) is an example of a program that is grounded in research findings [33]. The OEP is a personalized home-based intervention that includes exercises that promote strength, balance, and walking. The physiotherapist makes approximately five home visits across the period of the intervention; he or she also makes monthly phone calls to the participant to encourage adherence to the program. The patients are expected to independently exercise for a duration of 30 min at least thrice a week; in addition, they are required to walk for a duration of 30 min at least twice a week [34]. OEP has been found to reduce the number of falls and fall-related injuries, improve strength and balance, and maintain the fall-related self-efficacy of home-dwelling older adults [34].

However, the average uptake rate for simple exercise interventions among community samples is as low as 10% [14, 15, 28, 35–37], and few community dwellers who are prone to falls recognise their risk and prioritize preventive interventions [38]. Despite the abundance of research findings, the implementation of evidence-based fall prevention programs is still slow and limited [3, 4, 6, 36]; there is also evidence to suggest that these interventions do not reduce incidence of falling [3, 14, 15, 36, 37]. Therefore, researchers [3, 39–42] have underscored the need to bridge the gap between research-based knowledge and practice in the field of fall prevention in order to enhance the implementation of evidence-based fall prevention programs.

In a recent meta-synthetic study, health practitioners’ perceptions of falls and fall prevention were examined [3]. The authors concluded that health practitioners are at the forefront of fall prevention. However, van Rhyn and Barwick [3] concluded that research studies must be designed in such a manner that their findings can be readily and optimally applied in clinical practice.

Child et al. [4] have emphasized that it is necessary to address attitudes, beliefs, and behaviors at the individual, organizational, and societal levels in order to improve the implementation of evidence-based fall prevention programs [4]. Past studies have also explored the perspectives of healthcare professionals and community service providers on EBP [43, 44]. Their findings suggest that, when compared to earlier times, present-day physiotherapists are more likely to view evidence-based practice as an integrated patient-oriented enterprise [43]. However, these studies did not focus on evidence-based fall prevention programs.

Currently, there is limited information about the perceived barriers that may hinder the implementation of evidence-based fall prevention programs in the Norwegian and international healthcare context. To address this gap in the literature, we conducted a qualitative study to explore the factors that are perceived to hinder and facilitate the integration of empirical evidence and the OEP program among discharged hospital patients. The primary objective of the present study was to examine patients’ and physioterapists’ views and experiences about the important determinants of the application of research-based knowledge in fall prevention interventions. This paper is the first of a two-part report that addresses how OEP can be successfully implemented at the community level. This paper focuses on the individual characteristics of therapists and patients such as motivation, experience, research-based knowledge, skills, and respect for authority. The upcoming paper will focus on potential institutional barriers such as the organizational structure, leadership, human, material, and financial resources. It is necessary to examine the impact of these factors on the application of research-based knowledge in fall prevention. This study aims to promote knowledge translation by developing the evidence-based health services that are available to older adults and the health professionals who are engaged in clinical practice. It is expected that the findings will facilitate the delineation of improved strategies that can inform policy makers, educators, clinicians, future researchers, and older adults.

## Methods

### Design

The present study adopted a qualitative approach to explore and describe participants’ views and experiences [45]. According to Bottorff [46], qualitative inquiry has unique advantages that contribute to the exploration of the complex process of research translation. This study uses a phenomenological perspective, which explores how human beings make sense of experiences and transform those experiences into consciousness, both individually and as a shared meaning [47]. Thus, a phenomenological perspective incorporates the perceptions and feelings of people associated with what they experience, not merely the observations of the experience itself [48, 49]. The goals of this phenomenological perspective are to summarize individual experiences and to provide descriptions that include ‘what’ people experience and ‘how’ they experience it [50, 51]. We sought to understand the complexity of factors that influence the implementation of the community- and evidence-based Otago Exercise Programme for fall prevention through the perspective and experiences of patients and physiotherapists. Rather than starting with a specific theory, we inductively developed patterns of meaning through thematic analysis [52].

### Recruitment and participants

The last author recruited the physiotherapists from the hospital. The physiotherapists were provided with verbal and written information about the aims of the study and the associated data collection methods. In turn, the physiotherapist who worked at the geriatric ward recruited the patients as well as the municipality physiotherapists who administered the intervention. At the time of commencement of data collection, only four patients who were able to provide informed consent and had finished the intervention that was provided at their respective municipalities were eligible. Therefore, they were included in the present study sample; the sample also included physiotherapists who administered the OEP to the four patients. None of the clinical physiotherapists was involved in the research. The authors did not have any clinical role in this qualitative study.

A purposive sample of participants was recruited from an acute geriatric care ward in a large hospital in Norway [49]. Purposeful sampling involves selecting individuals or groups of individuals that are especially knowledgeable about or experienced with a phenomenon of interest, that contribute answering research questions [53]. In qualitative studies, 15 ± 10 participants is considered as a sufficient sample size to get information about the research phenomena and to yield a manageable amount of data [54]. Therefore, we aimed to recruit all physiotherapists and patients involved in performing the OEP and finished the OEP within 1 year. Hopefully, we would reach data saturation [49], in accordance with Malterud et al. [55] *“information power”*. The inclusion criteria for the geriatric patients were as follows: past experience of a fall, can independently rise from a seated position on a chair, and completed the OEP that was provided by their respective municipality after discharge from the geriatric department of the hospital. The exclusion criteria for the geriatric patients were as follows: a life expectancy of less than 1 year and a score that is lower than 23 on the Mini-Mental State Examination (MMSE). The patients’ ages ranged from 79 to 91 years (M = 84.5 years) and the duration of hospital stay ranged from 4 to 6 days. The demographic and functional characteristics of the four patients who participated in the present study are shown in Table 1. The inclusion criteria for the physiotherapists required that they were employed at a geriatric department in a hospital or in the primary health services in the municipality where the patients lived and had been referred to OEP. Furthermore, the four physiotherapists who facilitated the OEP for the four geriatric patients within community were also included in the study sample. Additionally, one hospital leader, two municipality leaders, and three physiotherapists who worked in the acute geriatric clinic were also included in the study sample because they play an important role in follow-up care that was provided by the municipality. With the exception of the compulsory school assignments during the bachelor’s (*n* = 8) and master’s programs (*n* = 4), none of the participants had been involved in research before. In total, twelve physiotherapists participated in this study (men = 2; women = 10). The ages of the twelve physiotherapists ranged from 23 to 66 years (M = 43.3 years). The demographic characteristics of the physiotherapists and leaders who participated in the present study are shown in Table 2.

**Table 1:** Demographic and functional characteristics of discharged hospital patients who participated in the present study

Patient ID	Gender	Reason for hospitalization	Marital/relationship status	Number of medication	SPPB point	BBS point	MSSE point	PCS-12 point	MCS- 12 point
1	F	Functional deterioration/decline	S	10	8,00	40	28,00	32,93	62,08
2	F	Congestive heart failure	S	2	6,00	42	25,00	38,88	45,50
3	M	Infection	P	5	11,00	42	27,00	39,45	40,76
4	F	Functional deterioration/decline	S	9	6,00	46	24,00	37,90	34,75

Note: *F* female, *M* male, *P* married/has a partner, *S* single, *SPPB* summed score for the Short Physical Performance Battery, *BBS* summed score for the Berg Balance Scale, *MSSE* summed score for the Mini-Mental Status Examination, *PCS-12* summed score for the Physical Mental Component Summary (Short Form-12), *MCS-12* summed score for the Mental Component Summary (Short Form-12)

**Table 2:** A profile of the physiotherapists who participated in the presented study

Physiotherapist ID	Gender	Year of graduation	Current occupational position	Type of interview
1	Female	1980	Section leader at a hospital	Focus group interview
2	Male	2014	Physiotherapist at a hospital	Focus group interview
3	Female	2008	Specialist in-patient physiotherapist	Focus group interview
4	Female	2007	Specialist in-patient physiotherapist	Focus group interview
5	Female	2012	Physiotherapist, municipality	Focus group interview
6	Female	1975	Leader, community health service	Focus group interview
7	Female	1991	Section leader intermediate care	Focus group interview
8	Female	1985	Specialist community physiotherapist	Focus group interview
9	Female	2014	Physiotherapist, municipality	Individual in-depth interview
10	Female	1991	Specialist community physiotherapist	Individual in-depth interview
11	Male	2012	Physiotherapist, municipality	Individual in-depth interview
12	Female	1989	Physiotherapist, municipality	Focus group interview and individual in-depth interview

### Data collection

Data were collected by the last author from September 2016 to September 2017 using focus group and in-depth interviews as the data collection methods. In total, eight individual in-depth interviews were conducted; of these, four were conducted with patients and four were conducted with physiotherapists. In addition, a focus group interview was conducted with the physiotherapist. One of the physiotherapists was individually interviewed, she also participated in one focus group interview.

### Measurements

To measure the functional characteristics and health-related quality of life of the patients, the following assessments were used: The Short Physical Performance Battery (SPPB) [56], Berg Balance Scale (BBS) [57, 58], Mini-Mental State Examination (MMSE) [59], and the Short Form 12 (SF-12) Health Survey [60]. These assessments were administered to the participants in their homes before the commencement of the OEP.

### Focus group interview

We used a focus group design as it allows participants to communicate, interact, and share their experiences with each other [61]. The method provides participants the opportunity to add further information to others’ statements. Furthermore, it facilitates spontaneous and informal discussions about the participants’ experiences and perceptions of the research phenomenon (i.e., implementation of EBP). The combination of focus group and in-depth interview might increase our understanding of the phenomena by comparing data in an iterative process and if the data were congruent, this can be seen as an indicator to enhance the trustworthiness of findings. Focus group with a phenomenological approach requires that each participant’s views and experiences are being respected. Nine physiotherapists and leaders (see Table 2) participated in the focus group interview (see Table 2), which was conducted at the hospital. Due to the Norwegian health care system organisation, this purposive sample was chosen in order to gain insight into various perspectives of the importance for the implementation of research-based fall prevention, e.g. recruitment of patients, implementation of new approaches at the workplace, as well as the clinical physiotherapists’ experiences with OEP. During the focus group interview, the physiotherapists discussed about the successful implementation of the evidence-based Otago fall prevention program within their community. We wanted to explore physiotherapists’ views and experiences on the factors that influence the implementation of the community- and evidence-based Otago Exercise Programme for fall prevention. In addition, in which the extent the program was compatible with patients’ preferences and physiotherapists’ clinical experiences. Furthermore, we sought to examine their meanings and experiences about the balance between research-based knowledge and practice-based knowledge, as well as the challenges that are faced by health practitioners and patients. This approach was considered to be appropriate because it can provide insights into different perspectives about if and how they consider OEP to be relevant. These perceptions will play an important role in the successful implementation of the fall prevention program. The focus group interview lasted for 90 min, and it was facilitated by the last author who is a physiotherapist and researcher in the field of fall prevention.

### In-depth interviews

In-depth interviews were conducted with patients and physiotherapists to explore the phenomenon research-based knowledge from the perspective of patients and physiotherapists. We anticipated that the information would enhance our understanding about the factors that facilitate or impeded the implementation of the evidence-based OEP.

Four patients (women = 3, men = 1) participated in the individual in-depth interviews and they spoke about their personal experiences with the OEP. These interviews were conducted in a dining room in the municipalities. In addition, four physiotherapists (women = 3, men = 1) were interviewed (Table 2). With the exception of one physiotherapist, who also participated in the focus group interview, all participants were interviewed only once.

Prior to data collection, we developed interview guides (see Additional file 1). For this purpose, we reviewed the literature on individual and organizational factors that either hinder or facilitate knowledge translation and EBP.

The researcher began the in-depth interviews by posing general questions and eventually presented progressively specific questions about their meanings and experiences with OEP and their beliefs and confidence about the successful implementation of the evidence-based Otago fall prevention program. Each interview lasted for 45 to 90 min, and they were facilitated by the last author, who presented herself as a researcher without any specific professional background. The physiotherapists who worked for the municipalities were interviewed by the last author in their respective offices.

### Context

In Norway, the healthcare system is predominantly state-funded and it is divided into types: specialist and primary healthcare. Primary healthcare includes home-based services and nursing homes, whereas specialist healthcare includes state-owned hospitals that are subsumed by four regional health authorities [62]. The acute geriatric ward is defined as a ward that has an independent physical location and structure, and is handled by a specialized multidisciplinary team that is directly responsible for the care of older adults with acute medical disorders [63]. After their discharge, all patients received home-based healthcare. In this article, home-based healthcare refers to the care that municipality-paid health workers provide to home-dwelling older adults. The healthcare that they provide entails multiple services that range from short-term rehabilitation to long-term assistance with activities of daily living and the advanced treatment of chronic and terminal illnesses [64].

### Ethical considerations

The present study was approved by the Norwegian Regional Ethics Committee (2015/1005 REK sør-øst D). The participants received written and verbal information about the study and their rights. Additionally, participants were guaranteed about the confidentiality of their responses, and they were reminded that their participation was voluntary. We emphasized that the physiotherapists could withdraw their consent to participate in the study without any adverse consequences to their employment. Furthermore, we assured the patients that they would still be able to participate in the exercise if they did not wish to participate in the study. All the participants provided informed consent, and none of them withdrew their consent at any point during the study.

### Data analysis

All interviews were audiotaped and transcribed by an external professional transcriber. Verbatim transcriptions were reviewed for accuracy and anonymized before they were distributed to the coauthors. We used the thematic coding technique in accordance with the framework that has been proposed by Braun and Clarke [65]. This is a six-step method that can be used to identify, analyze, and report patterns in qualitative data: (1) familiarizing the self with the data, (2) generating initial codes, (3) searching for themes, (4) reviewing the identified themes, (5) defining and naming the themes, and (6) preparing the report. Subsequently, we examined the ideas, conceptualizations, and assumptions that underlay the expressed content [65]. In order to preserve variability, encourage reflexivity, and establish credibility, all authors analyzed the data independently. All the authors are professionally trained educators and health professionals who have a background in physiotherapy and nursing. The resultant themes and subthemes were consolidated based on consensus among the researchers. Additional file 2 shows a summary of the emergent subthemes, main themes, and overarching themes.

To enhance trustworthiness and limit the potential threats to validity, we employed the criteria for “trustworthiness” that have been described by Lincoln and Guba [66]: credibility, transferability, dependability, and confirmability. Credibility was ensured through open-ended questioning, prolonged engagement with the data, and articulation of a detailed description of the methods. Transferability was achieved by providing an in-depth, detailed, and descriptive analysis of the data and by quoting participants’ responses to substantiate the findings. With regard to dependability, the transcriptions were reviewed several times, and they were checked and coded by all authors; further, interpretations were arrived at based on consensus among all authors. Confirmability was achieved by substantiating each emergent theme with rich quotes that were extracted from the participants’ responses.

According to Berger [67], the position and reflexivity of the qualitative researcher are of paramount importance at all stages of the research process. Accordingly, the researchers’ professional background (i.e., healthcare personnel in the field of physiotherapy and nursing) and clinical experience (i.e., in community healthcare) may have affected the data collection and analytic procedures. Specifically, the researcher who conducted the interviews was familiar with the “language” of the research context and could therefore address certain topics or pose follow-up questions during the interviews. This might have influenced both the quantity and quality of the data in a positive manner (i.e., enrichment of the data). However, there is also a risk that the researcher might have overestimated the between-participants similarities and consequently overlooked individual differences in experiences; this may have impeded the discovery and construction of new knowledge [68]. To avoid this, the researchers maintained a constant sense of awareness about how their preconceived notions may affect the study findings both during the interviews and data analysis. The emergent subthemes, main themes, and overarching themes are presented in additional file 2.

## Results

The thematic analysis yielded five subthemes and two main themes that were integrated into one overarching theme: *A dynamic integrative process: successful implementation requires researchers, patients, clinicians, and organizations to modify established perceptions, behaviors, language, and practice into routine behaviors.* The physiotherapists and geriatric patients highlighted the relevance of research-based knowledge in addressing the challenges that are encountered in clinical practice. Consequently, the patients felt safe and experienced its positive effects, and it enhanced their ability to manage the demands and requirements of daily life. The results suggest that knowledge translation does not happen automatically. Instead, mutual understanding among the different user groups necessitates the process of negotiation. Research-based knowledge is not universal, and it cannot be transported from one context to another; therefore, it can only be adapted and translated. However, the application of research-based knowledge does not equate to the following: *filling up empty jars* or *flows from the expert to the non-expert as water through a tube.* Neither the physiotherapists nor the patients are *tabula rasa*. Furthermore, physiotherapists considered user-friendly language and mindsets to be important for the successful implementation of EBP. In the following sections, the two main themes and their corresponding subthemes are presented along with selected quotations from the transcribed material that illustrates the findings. The subtitles serve as a summary of each theme.

### The researcher’s role and position in the implementation process

In this section, the main theme and the two subthemes are presented: 1) The researcher was full of himself and 2) The “bottom-up” approach: collaborative interests.

The physiotherapists in both the focus group and in-depth interviews discussed their perceptions of researchers and the role that they play in the EBP. They agreed that researchers’ attitudes have significant implications for the implementation of the OEP. Further, all the participants in both the focus group and in-depth interviews observed that it is important for researchers to demonstrate a sense of interest and humility toward the clinical field and with regard to their contribution to knowledge development. They argued that researchers are primarily concerned about the achievement of their own research goals; consequently, they forget that research benefits both the practitioner and the patient. Regarding OEP, the participants in both the focus group and in-depth interviews noted that it was relevant for clinical practice. Further, all the patients appreciated the fact that the intervention that they participated in was based on empirical evidence.

#### “The researcher was full of himself”

The participants in the focus group observed that researchers, therapists, and patients play different roles and occupy powerful positions that pertain to the application of research findings in the field of fall prevention. The patients believed that EBP ensures high-quality treatment. The physiotherapists also highlighted that the researchers readily played the role of a leader. They also emphasized that the acumen and involvement of physiotherapists as well as the knowledge and expertise of researchers and patients play a crucial role in the cooperative exchange of knowledge that is necessary for the successful implementation of evidence-based practice. The participants in the focus group observed that there is a disparity in power and knowledge across the three groups, namely, researchers, health professionals, and patients.

Some of the participants in the focus group considered the perceived role of an expert that researchers play to be an important challenge that impedes the implementation of EBP. They reported that researchers often approach clinical practice with ready-made solutions and definitive answers. One focus group participant stated:

*The researchers have to be more ordinary and not full of himself and his research project, as the research communication becomes inadequate*

To overcome this challenge, some physiotherapists from the in-depth interviews suggested that the researcher must move away from one-way communication and toward dialogue with practitioners. It is also important for researchers to be *down to earth* and respect the experiences of the clinical practitioners. They believed that the researcher’s interest, openness, and ability to reflect and create egalitarian relationships are factors that are essential for one to successfully approach the field of clinical practice. One focus group participant explained this point in the following manner:

*If the researchers are to succeed with implementation or if we are to succeed with implementation, the researchers must show that they acknowledge the other and have respect for others’ views. They must not always be concerned about persuading. They must be willing to compromises, for example, the field of practice has much important knowledge to succeed with the implementation. Being willing to more active listening, try to understand the other's perspectives and professionalism. It’s about a common understanding*

However, it may seem that researchers who examined OEP have succeeded in both communicating and understanding the complex area of clinical practice. As one of the physiotherapists who participated in the in-depth interviews noted:

*OEP represents good physiotherapy practices. It is a system where you work according to some principles. I perceive that it is based on experiences from people who are concerned that it should work in practice.* (Physiotherapist ID 11)

The feedback that was provided by the four patients suggested that the researcher’s elevated authority and powerful voice may play an important role in how patients experience EBP. Patients considered the OEP to be credible. They believed that treatments that are grounded in research are effective and of a good quality. One of the patients made the following remark:

*...research provides a sense of security , and I get more confidence that it can do something for me in my situation. You have to exercise. It stands in the newspapers periodically. It is strange that no more does it, when the effect is so good. I feel it on my body, and I get more drift and motivation. It’s a good thing. I don't know much about research, but I experience it as a security.* (Patient ID 3)

#### The “bottom-up” approach: collaborative interests

All the physiotherapists were concerned about the means by which researchers can bridge the gap between research and practice. The physiotherapists described a desire for closer ties between research and practice. Similarly, the patients noted that the OEP was compatible with their needs and preferences. The participants in the focus group also reported that researchers tend to be insensitive to the role that patients and physiotherapists play in the implementation process because of their general lack of interest in clinical practice. One of the physiotherapists who participated in the focus group made the following observation:

*The researchers have to be curious about the patients’ challenges and the field of practice knowledge. Show that they actually are interested*—*not just pretend*—*to achieve what they think is important. They actually have to ask the patients and practitioners about their understanding and perception or what matters to them.* (Focus group interview)

The participants in both the focus group and in-depth interviews believed that a sense of interest in the current affairs of clinical practice will provide researchers with knowledge and better preconditions that can aid the application of research-based knowledge in clinical practice. This was described as a process of co-creation whereby one arrives at a consensus about the recommendations that pertain to the use of research-based knowledge. This type of a collaborative approach was considered to be essential to the development of resources that are useful to physiotherapists as well as readily implementable in clinical practice. They believed that, in order to succeed, researchers must possess sufficient knowledge about the needs of the respective field of clinical practice. The researcher must also understand that users are constantly forced to act in accordance with a service framework and established routines that may challenge and adversely affect optimal decision-making. One of the focus group participants provided the following explanation:

*Implementation is about a change or a process of transformation that both affect and are affected by many different practitioners and factors at different levels in the organization or service. The researchers must be humble and take into account the complexed interplay between factors who affect the implementation to satisfactorily understand and analyze the implementation processes and their outcomes and results.* (Focus group interview)

As users of research findings, the physiotherapists wanted their voices and experiences to form the basis for further mapping of the need for competence in the implementation of empirical evidence in clinical practice. The research study has to be adapted in accordance with the target group or intended purpose. One of the physiotherapists from the in-depth interviews contended that cooperation will facilitate the implementation of empirical evidence in clinical practice and clarify the rationale that underlies EBP. One physiotherapist who participated in the focus group made the following remark:

*I think it’s nice that researchers become more interested in what the practice field believes and their experience base. I think that in itself will facilitate the implementation of research in practice. We would like to use something that certainly works.* (Focus group interview)

The physiotherapists emphasized the importance of using patient participation in research to enhance the relevance of the findings to clinical practice. They believed that the user’s experiences and perceptions are crucial because they allow one to tailor the intervention to the intended audience. Regarding the OEP, this evidence-based intervention were referred as logical and understandable to both the physiotherapists and patients. One of the physiotherapists who participated in the in-depth interview described this point as follows:

*I think it seems logical—the intervention is not difficult to get accepted from the patient. In my opinion, it is not a big gap between clinical practice and Otago. I understand the rationale of the program. It represents good physiotherapy practice. It provides a system to work after ... provides some principles.* (Physiotherapist ID 11)

All patients who participated in this study expressed a satisfaction with the OEP and found the exercises to be compatible with their daily activities. This suggests that the researchers who designed the Otago are genuinely interested in clinical practice. Indeed, one of the patients said:

*I've got better balance. I notice that I walk steadier. I am steadier when I get up and I become more confident. I do more housework because I am not that apathetic anymore. I simply have more energy.* (Patient ID 4)

One of the patients articulated his perspective on the importance of research on fall prevention interventions in the following manner:

*Although I do not know how it is done, I think that research improves the services ... research means that the service is safe and creates trust ... I think research means that the intervention is quality assured to some extent. I have confidence in the researchers. They have authority and hopefully they have knowledge, so we can trust the results.* (Patient ID 1)

### The tension between research-based knowledge and clinical practice

This theme pertains to the view that research findings are typically perceived as a “guideline” for clinical practice. The physiotherapists identified various barriers that impeded the application of research-based knowledge in clinical practice. However, this does not seem to affect patients. However, the patients who participated in the present study considered the intervention to have been individualized to meet their unique requirements. This main theme subsumes three subthemes: 1) *the need for reflective or critical uncertainty in practice and research*, 2) *creating mutual language: translation leads to transformation,* and 3) *the patient’s lived experiences and the clinician’s knowledge*. Each of these subthemes is presented in the following sections.

#### The need for reflective or critical uncertainty in practice and research

The physiotherapists reported that their clinical decisions are based on complex assessments. They believed that research findings do not represent the absolute truth but always entail an element of uncertainty. The physiotherapists also noted that some of their colleagues were skeptical about the applicability of research in clinical practice. They referred to conflicting research findings and diverse research skills. Further, they contended that the element of uncertainty is a result of probability calculations; in other words, research findings do not represent the absolute truth. Different perspectives are central to the successful application of research-based knowledge in clinical practice. This is illustrated in the following claim that was articulated by one of the physiotherapists who participated in the focus group:

*We need to be thrilled —don’t be afraid of disagreement. It is through dialogue we create attitudes to what to be implemented and these attitudes form the basis for how others relate to us and give us feedback on our attitudes and changes in our subject perspective ... the practitioners must be aware of any strengths and weaknesses of research.* (Focus group interview)

The physiotherapists from the in-depth interviews stated that research must be integrated with clinical experience, as well as it has to take into account the needs, resources, and desires of the individual patient. The application of research findings, even those that are of a so-called “high quality” or meet the “golden standard,” must be seen in the context of person centred care. Furthermore, physiotherapists noted that the various sources of knowledge (i.e., experts’ statements, research findings, guidelines, and courses) can contribute to the professional development of clinical practitioners. Collaboration among colleagues was highlighted as an important resource and a source of knowledge. During such interactions with others, knowledge is exchanged. However, the process might also reveal one’s attitudes towards research. These interactions can also help individuals (including researchers) provide feedback and consequently modify the perspectives of the receiver. The interaction and the associated uncertainty offer practitioners the opportunity to gain new knowledge by collaborating with researchers, colleagues, and patients. Such a contention is exemplified in the following contention that was provided by one of the physiotherapists who participated in the in-depth interviews:

*We have to accept that we understand things differently and that somebody sometimes perceived that they do not fully understand what research is about. Ambiguity and acceptance of different degrees of understanding provide the opportunity for negotiation.* (Physiotherapist ID 9)

One of the patients also described how her physiotherapist individualized the intervention to address the challenges that she faced with regard to her sense of balance in the following manner:

*They have to know what are relevant interventions and tailor this to the elderly ... They must be good communicators ... I think the exercises were very nice and fall preventive ... balance exercises strengthen the leg muscles and generally the legs and body. I think it’s terrific.* (Patient ID 1)

#### The patient’s lived experiences and the clinician’s knowledge

A majority of the physiotherapists perceived EBP to be a solution that lies at the intersection of their own experiences and research-based knowledge, and the patient’s needs and desires. The participants who contributed to the focus group discussions described this trinity as a fusion of knowledge. Indeed, one of the physiotherapists made the following remark:

*Evidence-based practice is the expression you want the practice to be adapted to best scientific evidence as a foundation to decision-making, that is it is about both research, professional knowledge, and the patient's desires and the context. You must get a fusion of knowledge where all the participants contribute, that is researcher, practitioner, and user, that is the patient.* (Focus group interview)

The participants in the focus group stated that neither the physiotherapists nor the patients are *tabula rasa*. They were especially concerned about the patient’s life experiences, wishes, and goals, and believed that this should be the foundation of practitioners’ active involvement in their treatment and interventions options. Accordingly, one of the participants in the focus group made the following observation:

*We know that it is not about filling up empty jars ... but it has to be linked to the users’ desires and preferences ...* (Focus group interview)

Several physiotherapists contended that research studies should be cognizant of the challenges of clinical practice; in other words, the application of research findings must be contextualized. In addition, patients, relatives, and therapists have different desires and preferences; additionally, empirical findings play an irreplaceable and crucial role in successful implementation. The intervention that was examined in the research study was considered to be a prototype that had to be adapted to meet the unique needs and requirements of the individual. Such a contention is exemplified in the following observation that was made by one of the participants in the focus group:

*We do not believe that research flows from the expert to the non-expert as water through a tube. It has to be transformed and adapted to the user. The research is generalized and there are few patients that represent the norm. It is a prototype, which has to be modeled within the interaction between patient and practitioner.* (Focus group interview)

One of the physiotherapists who participated in the in-depth interview contented that the patient’s intentions regarding treatment have significant implications for treatment implementation in the following manner:

*Change in behavior is about the patient taking his action and its consequences into consideration. The patients have to have an opinion about it, make decisions about how they want to live their lives. Understanding is, therefore, important. We have to respect the person's autonomy while exploring the opportunities for behavioral change. Lack of compliance to our recommendations may also be because the person does not realize that there is a problem. We need to think about the underlying psychological factors that lead or hinder a change and help even more people manage to implement the intervention.* (Physiotherapist ID 10)

Furthermore, she observed that the patient must have faith in one’s own ability to meet the requirements of the training program. The ability of the patient to adapt to the requirements of the training program is therefore crucial for success. One physiotherapist stated:

*The Otago program requires confidence that the patient can succeed with the performance—it must be adapted to the individual patient. They must have faith that the program can bring them something positive and that they are able to do it.* (Physiotherapist ID 10)

The patients noted that the training enhanced their physical capabilities and quality of life*.* The combination of the OEP and clinician’s knowledge had significant implications for their functioning. Thus, the OEP provided the energy that they required to meet the requirements of daily life. In this regard, one of the patients made the following observation:

*When I walk, I notice the effect … that it had an effect at all! When I dress myself, I am steadier than before I started to exercise. Everything I do at home goes well now! I’ve got a new life. Hope it lasts.* (Patient ID 4)

#### Creation of a mutual language across different users: translation leads to transformation

Several physiotherapists highlighted the need for a common language across researchers, practitioners, and patients. As professionals, they found the terminologies that were used in the empirical literature difficult to understand. Accordingly, one physiotherapist made the following comment:

*The research language is not always easy to understand. It can be experienced as a tribal language and if you have a basic education in physiotherapy or nursing you have not learned the tribal terms as those who research.* (Focus group interview)

Furthermore, the cognitive and affective impact of the use of obscure terminologies may adversely affect the attitude of the physiotherapist, the recipient of the research and consequently serves as a barrier to implementation. One physiotherapist during the focus group interview said:

*The words we choose create feelings, thoughts, and actions through the meaning they give to each of us. This can constitute closures—we do not automatically understand the terms and what is included.* (Focus group interview)

The language that is used when interacting and communicating with the patients was also considered to be a barrier to implementation. Specifically, they contended that research-based knowledge should be presented in a language that both patients and practitioners can understand. In this regard, one of the physiotherapists made the following observation:

*The terminology must take into account that other people understand things differently ... the research-based knowledge must in some way be translated into practice.* (Physiotherapist ID 12, female)

In addition to terminological challenges, the guidelines are also defined differently. The information is perceived to be complex, and this perception eventually leads to poorer use and implementation. Typically, users are not familiar with guidelines of an intervention program, and the participants in the focus group indicated that there was also a lack of communication and distribution to the users. One of the physiotherapists believed that treatment implementation entails the exploration of the factors that facilitate and inhibit the translation process between the practitioners and researchers. One of the physiotherapists who participated in the in-depth interviews described the means by which the challenges that pertain to research language can be solved. Additionally, he also articulated the means by which practitioners can contribute to the incorporation of research into practice. Specifically, he made the following comment:

*I also see that we can contribute as ‘translators’ in the research communication of research to practice. We, physiotherapists, due to our participation in this project become slightly ambassadors for the ongoing research-based methods and help to make the knowledge or experiences produced within the project accessible and understandable to other practitioners and decision-makers.* (Physiotherapist ID 11)

Another physiotherapist underscored the importance of the physiotherapist’s communication skills in establishing the relationship between the patient and therapist; she also contented that it affects the level of motivation and effort that the patient invests in the treatment. As illustrated in the following excerpt, the physiotherapist described the patient-therapist relationship as a trust-based relationship:

*It is important to acknowledge the elderly as an expert on their experiences and experiences with communication and relationship understanding ... I must be responsive and try to understand the patient's world and experience background. Try to take the patient's perspective and not just shape the patient from what I think.* (Physiotherapist ID 9)

In addition, research-based knowledge has to be communicated to the patient in a suitable manner. Specifically, patients should be able to understand the message. One patient recollected that her physiotherapist was proficient in adapting and communicating research-based knowledge in a simple and comprehensible manner. Consequently, the patients felt that they were treated as unique individuals, and in this manner, the physiotherapists allowed them to stay in control of their own recovery process. The patients reported that the OEP was delivered in a respectful manner and that it was individualized to meet their unique needs. They were allowed to negotiate, and they were provided with alternatives; in this manner, the therapeutic relationship allowed them to feel empowered to take health-related decisions. In this regard, one patient made the following observation:

*She sees you, she answers your questions in a simple way, so I understand it ... she knows a lot and doesn’t overrule you, but talks about the most important things.* (Patient ID 4, female)

Another patient said:

*The way you are feeling welcomed is very good. They are so gentle and you have the feeling that you are so welcome. They asks questions about how I have experienced the exercises or social things I had told I would attend and they showed that they were interested in my answers.* (Patient ID 2)

The patient provided the following additional comments:

*My physiotherapist was very clever and attentive. We focused on balance training, walking training, and strength training. I was tired but did not want to give up. He praised me and said, ‘Now you were good!’ He gave me advices and followed me well. Asked about how did it go etc. It gave me a lot. He discovered positive changes such as that I managed several repetitions or more weight or resistance or that I had better quality of what I did ... did not seem so tense. I became friends with my body in a way. I got confidence ... praise motivates and gives hope. Hope is important. The training supports hope.* (Patient ID 2)

## Discussion

We believe that this is the first study to focus exclusively on user views on the important determinants that influence the implementation of the community- and evidence-based OEP for fall prevention. The findings of the present study can help bridge the gap between research and practice. The feedback that was provided by the patients and physiotherapists suggested that the OEP can guide clinical practice. Specifically, the participants (i.e., both patients and physiotherapists) noted that the research-based knowledge that the OEP entails is relevant, powerful, effective, and safe for use when it is adapted to meet the unique needs of the patient and communicated using user-friendly language. The findings underscore the importance of combining and integrating the different sources of knowledge and experience that are available in the clinical context of: 1) the patients’ values, preferences, and experiences, 2) clinical expertise, and 3) research-based knowledge. These findings are resonant with current opinions that fall prevention interventions must be grounded in empirical evidence.

EBP is a term that is used in clinical practice, and it is informed by research, clinical expertise, and patients’ needs, values, and preferences, as well as the role that they play in health-related decision-making [43, 69–72]. Both patients and clinical experts (physiotherapists) found the EBP of OEP to be relevant and useful. According to the participants, bridging the gap between research-based knowledge and practice leads to improved patient-perceived outcomes. In addition, enhancing the self-confidence, and critical thinking and decision-making skills of practicing physiotherapists will be significantly beneficial to them. Perraton et al. [73] noted that assisting physiotherapists’ application of research-based knowledge in clinical practice can help enhance the quality of clinical practice and encourage lifelong learning and professional progress.

EBP seems not to be what the researchers have documented. According to our participants, researchers do not focus on EBP. Additionally, they reported that it is important to adapt research-based knowledge to meet the unique demands of varying clinical contexts. Our patients reported that the OEP addressed their needs. This raises the question that was proposed by Ritchie [74]: “How far can we go in adapting the knowledge to the local context?” The physiotherapists stated that there is a certain degree of tension between standardization and individualization of the knowledge that has been derived from clinical randomized trials. However, they also acknowledged that the knowledge that has been yielded by randomized controlled trials should be adapted to meet the unique needs of the patient. Research-based knowledge must serve as a template that must be customized in accordance with the needs of the patient and the experiences of the physiotherapist. Our patients believed that the OEP enhanced their capability to cope with the demands of daily life. The integration of practice-based and research-based knowledge within the context of clinical practice has been addressed by Nilsen and Eilström [75]. Healthcare providers must have the requisite skills and expertise to apply research-based knowledge and customize based on the unique requirements of the local context. The interpersonal and interactive nature of physiotherapy requires different types of knowledge that pertain not only to diagnosis, prognosis, and a biomedical understanding of human anatomy and physiology but also about the experiential dimensions of living with impairments and receiving physiotherapeutic treatment [76]. The integration of different types of knowledge and the practice of physiotherapy has been strongly emphasized in recent times because there is international and national emphasis on the involvement of patients in therapy and healthcare [77]. The feedback that was provided by all four patients indicated that their preferences had been taken into account during the implementation of the OEP. Further, all the patients were satisfied with the quality of the interventions. The World Health Organization defines good-quality healthcare as one that is effective, people-centered, equitable, integrated, and efficient [78]. The extent to which the quality of healthcare can be considered to be “good” is contingent on the extent to which the service is able to meet the needs of its users [79] as well as the extent to which it can be adapted to address the expectations and preferences of the patient [80]. The findings of the present study suggest that the needs of the patients were met by the OEP.

The usual search for explanations and solutions that can address the gap between research and practice entails an analysis of the means by which the results can be communicated to the practitioners in a more efficient manner [81]. In contradistinction to such a perspective, the physiotherapists who participated in our study emphasized that researchers must ask patients and practitioners about what matters to them*.* In accordance with the contention that has been proposed by Green et al. [81], the participants stated that the research-based knowledge that is directly derived from the respective research studies is not readily usable; instead, it has to be adapted to meet the unique needs of the practitioner and patient. The physiotherapists stated that EBP serves as a novel lens through which one can critique practice, consciously analyze problematic situations, examine assumptions, research the available options to justify their use in practice, and find ways to effectively apply research-based knowledge.

Jones et al. [82] have contended that research and practice guidelines were never intended to be communicated in a prescriptive manner to patients with unique physical features, pain-related problems, and disabilities. A key challenge that faces the field pertains to the means by which clinicians’ application of research-based knowledge can be facilitated [82]. The importance of critical reflection during the process of applying research-based knowledge was emphasized in both focus group interviews and in-depth interviews. The participants also underscored the importance of professional judgment and sound clinical skills. Further, the physiotherapists contended that research findings will never be a perfect representation of the absolute truth. Therefore, EBP must also be subjected to continuous dynamic changes based on the demands of a given context. Green et al. [81] claimed that one might reasonably conclude that science will always have to bridge the gap that alienates it from practice. As long as research-based knowledge is generated within academic contexts that emphasize scientific control for the sake of internal validity, external validity will not receive the due attention that it requires [81]. However, according to our participants, the OEP has a high level of external validity.

In contrast to our findings, Haas et al. [83] found that physiotherapists believe that evidence-based fall prevention exercise interventions lack clinical applicability. In the present study, the physiotherapists’ critical assessment of the applicability of the OEP among those with cognitive impairments is consistent with this finding. However, overall, the physiotherapists and patients found the OEP to be useful, relevant, and transferable to the intended target group. This finding is supported by the fact that the OEP is the most widely used fall prevention program [84]. Its effectiveness with regard to fall prevention has been confirmed by several researchers as well as the present findings. Furthermore, the patients’ feedback suggested that the OEP helped them manage the demands of their lives and that it was easy to understand; hence, they were extremely satisfied with the program.

The physiotherapists proposed that research studies should be based on an in-depth understanding of clinical practice, including the challenges that are faced by the deliverers and recipients of the interventions. The participants underscored the importance of ensuring that research-based knowledge is adapted to the unique limitations of clinical practice. According to Shayan et al. [85], there is a communication gap between academic researchers and clinical practitioners in the field of nursing. The participants emphasized the need for closer ties between the academic and the clinical practice environments; they also emphasized the importance of practice-oriented research; past studies have reported similar findings [83, 86].

The present findings resonate with Foucault and Gordon [87] propositions that knowledge is imbued with power (i.e., research), and that in turn power produces knowledge (i.e., EBP). In this manner, knowledge is prioritized and promoted as the most important prerequisite to understanding the field. Some participants suggested that researchers must acknowledge others and show that they have respect for others’ views; in other words, they must not seek to promote only their own opinions. According to Foucault [88], power can be understood as a productive force. Specifically, power is not “possessed” by certain people; instead, it is demonstrated through peoples’ actions and attitudes in everyday life [87]. The physiotherapists also emphasized the importance of creating a *common understanding*. This finding is in accordance with the contention of Kuper and Whitehead [89] that power creates knowledge, and that the language that is used to describe and talk about a topic (discourse) influences the thoughts and spoken content of people who belong to a given field. Furthermore, certain types of knowledge, and ways of thinking and talking about a particular topic are more dominant than others [90]; it is important to be conscious of these differences. During discourse, power guides people to think and act in certain ways, and privileges some types of knowledge (e.g., research-based knowledge) over others (e.g., practice-based knowledge, the preferences of the users/patients) [88]. This suggests that the manner in which people think about and approach a particular topic (e.g., evidence-based fall prevention interventions) are structured by the discourses that are used to describe and talk about it [88, 91]. The participants also focused on the importance of transforming research-based language into a language that is comprehensible to clinicians and patients. In the most elemental sense, power is the ability to influence what happens to oneself; therefore, the manner in which one speaks is of great importance. Patients require knowledge as well as the power to participate in their health-related decision-making process [92].

Our findings suggest that physiotherapists want researchers to adopt other roles; specifically, our participants commented that *“a researcher should not be full of himself and his research*.*”* The definition of an expert and the corresponding role distribution has also been discussed in a systematic review that was conducted by Child et al. [4]. The researchers adapted the methods for community use, and they examined the different social and cultural factors that influence the use (and acceptability) of assistive devices, types of exercises, and fatalistic attitudes toward falls that are evident across different communities. Furthermore, Child et al. [4] observed that the ways in which fall prevention interventions are reported entail substantial methodological challenges that often inhibit the application of the respective research findings in practice. Another study that was conducted by Ballinger and Payne [93] also showed that academic expertise has important implications for treatment as well as the trust and faith that the patient has in the therapist. With respect to the application of research-based knowledge in clinical practice, the physiotherapists and patients contended that success requires a mutual understanding of each other’s knowledge, namely, life lessons, professional knowledge, and clinical experience.

Adopting this particular perspective (i.e., the influence of power on knowledge that pertains to research and health services) allows one to critically reflect on how their thoughts and actions are influenced by the specific type of knowledge that they rely on. Issues of power were evident in our findings, and it pertained to the (communication) gap between researchers and practitioners. The responses that participants provided underscored a mismatch in the different roles that the application of research-based knowledge necessitates. In our study, physiotherapists referred to researchers as experts, and practice was described as a type of power. Power has been conceptualized as a resource and as the capacity to act, but researchers acknowledge that this concept is debatable [94, 95]; however, this did not appear to have a negative impact on patients. On the contrary, physiotherapists perceived themselves as the communicators of research-based knowledge; this ensures that the research findings are conveyed to the patient with confidence and credibility. This suggests that physiotherapists who serve as the communicators of research findings serve as an authority figure and as a professional expert whom the patient trusts. Further, the patients also reported that the therapists communicated the research-based knowledge to them in an understandable and humble manner; this promoted their sense of meaning in life.

The physiotherapists who participated in the present study repeatedly mentioned that their ability to adapt and explain the exercises to the patient is important for the successful implementation of the OEP. Similar findings have been reported in a previous study [86]; specifically, negotiations regarding the research-based knowledge has also been found to frequently occur across different patient groups [96]. This underscores the importance of integrating clinical expertise, patient preferences, and research findings. Physiotherapists highlighted the importance of user involvement as a part of their clinical practice. This approach has been emphasized by Mackenzie [86], and it describes the aspect of EBP that compromises patient preferences. Snöljung and Gustafsson [43] found that, when compared to earlier times, present-day physiotherapists viewed EBP as an integrated and patient-oriented enterprise; this contention corresponds with the feedback that was provided by the patients who participated in the present study. This emphasizes the importance of individualized and tailored interventions. Consistent with our participants’ statements, past studies have reported that joint decision-making and negotiations enhance the client-therapist relationship across a variety of clinical settings [97], including falls prevention [86, 98]. Furthermore, the patients who participated in our study contended that the physiotherapist’s presence and therapeutic sensitivity to the individual are essential for the successful implementation of the OEP. The results of a study on clinical reasoning that was conducted Ahlsen et al. [99] showed that the focus of clinical practice is the care that is provided to the individual. As such, practice involves the exploration of the extent to which knowledge that is derived from groups of patients are relevant to a particular patient. In our study, the patients believed that the OEP was relevant. However, as demonstrated by Ahlsen et al. [99], the process of applying different types of knowledge sources (e.g., research-based knowledge, patients’ narratives, clinical experience) necessitates interpretation. In this regard, the EBP movement has prompted a growing focus on clinical reasoning and decision-making among health professionals (i.e., including physiotherapists), and this trend corresponds to our findings.

A clinician must be sufficiently skilled to address the unique values and needs of the patient using the best available empirical evidence. According to the patients who were interviewed in the present study, the clinicians were able to successfully tailor the OEP in such a manner that it meets the unique values and needs of the patient. The physiotherapists focused on the importance of understanding EBP as a process that entails the following steps: knowing which clinically oriented research question to formulate, articulating how one can find the best available treatment, and developing a strategy to critically appraise the validity and applicability of the intervention in a given clinical situation. Regarding the OEP, one of the physiotherapists reported that he *understood the rationale of the program.* Others underscored the importance of being able to interpret research-based knowledge in new contexts. The physiotherapists also stated that the transfer of knowledge is not akin to *filling up empty jars.* Further, one of the physiotherapists observed that *research does not flow from the expert to the non-expert like water through a tube. It has to be transformed and adapted to the user as well as to the clinician’s knowledge.*

There are several definitions of best practice such as “optimal level of care” [100, 101] or “optimum quality of care” [102] that correspond to our patients’ views. Furthermore, Schreiber et al. [103] have stated that, as per the definition of EBP, clinical decision-making should be guided, at least in part, by the best available research. To apply research-based knowledge by means of knowledge translation, physiotherapists need to read, interpret, and understand the literature that has been presented using research-oriented and statistical language. Accordingly, the physiotherapists were concerned about the adaptability of research-based knowledge. They also expressed concerns about the terminologies and language that researchers used, as well as the production and interpretation of the research results. According to the results of a recently published systematic review [85], nurses considered a lack of familiarity with the terms that are used in research articles, and difficulties in appraising research findings and translating them into practice to be barriers that impeded the implementation of EBP. In another recent publication, Snöljung and Gustafsson [43] noted that the barriers that physiotherapists encounter in their implementation of EBP may be related to a lack of expertise and a limited understanding of scientific research. Further, in accordance with our findings, previous studies have also shown that most physiotherapists are interested in improving their EBP skills and that they have a positive attitude toward EBP [104–108]. In our study, the patients and municipality physiotherapists who conducted the OEP considered the figurative distance between the OEP and their own clinical practice to be small, and they believed that the program was compatible with the patients’ reported preferences. The physiotherapists also reported that the OEP complied with best practices in physiotherapy and the pertinent guidelines. The patients endorsed this perspective, and they added that the research-based knowledge augmented their confidence in the treatment that they had been provided. According to our participants, EBP serves as a new lens through which one can question clinical practice. It allows one to consciously analyze problematic situations, examine assumptions, identify research options that justify changes in clinical practice, and delineate strategies to effectively implement the required changes.

### Strengths and limitations of the present study

The goal of qualitative research is to enhance one’s understanding of the phenomenon of interest [45]. The present findings delineate what physiotherapists and patients consider to be the factors that influence the implementation of the evidence-based OEP for fall prevention. The feedback that was provided by the users provides important information that can be used to individualize the intervention in such a manner that it addresses the unique needs of the patient.

With regard to the limitation of the study, we recognize that including a larger number of participants, who represent different geographical regions would yield a wider range of perspectives on the important determinants that influence the implementation and application of EBP in fall prevention. Maybe a larger number of patients might have added more information, however, Creswell and Poth [49] ranges are a little different. They recommend between five and twenty-five interviews for a phenomenological study, while Morse [109] suggests at least six. Although, we had a small sample size, it did represent leaders at hospital and municipalities, as well as the physiotherapists and patients who had participted in OEP, which in the literature are described as important for the implementaton of evidence-based practice [4]. As a qualitative study, with a small sample size and that used purposive sampling, generalizability to other populations is cautioned. Generalizing of qualitative findings is usually not a goal of qualitative research, but rather transferability. To address this, we aimed to provide rich and detailed descriptions of the participants’ views and experiences, as well as the context of this study [66, 110]. Thereby, the present study make a valuable contribution to the field by bridging the gap between research and practice. Researchers’ preconceptions influence the research process [49]. The authors of the present paper have a background in physiotherapy and nursing. Throughout the research process, we were conscious that our existing professional knowledge and backgrounds can impact our data interpretations. Therefore, we tried to interpret the data as objectively as possible, and we have strived to provide a transparent description of the research process (i.e., from data collection to interpretation). The involvement of multiple researchers with different professional backgrounds may have also strengthened the design of the present study because they complemented and contested each other’s statements. In addition, the interviewer did her best to make the participants feel comfortable, and also listened to their stories with empathy and attention.

Observational data collection techniques (i.e., in addition to interviews) can contribute important information because knowledge is manifested through practical actions, and consequently, we can “discover” what we know by observing how we act [111]. Interview data do not represent the absolute truth; instead, they are highly dependent on subjectively chosen perspectives. Accordingly, the present study conducted using a relatively small group of sixteen participants; however, this sample size is in accordance with the recommendations that it is ideal to analyze ten to fifteen interview transcripts at a given time [112]. The study was conducted with authority figures and stakeholders in the field of fall prevention, across three municipalities in Norway. These respondents were chosen because they were important stakeholders and decision-makers in their respective municipalities; consequently, their perspectives have implications for the delivery of physiotherapeutic services in community-based fall prevention interventions [113]. This suggests that similar perspectives may prevail in other physiotherapeutic settings (at least those that consist of stakeholders and decision-makers who play a role in the Norwegian physiotherapeutic services); however, there may be differences in the extent to which the themes that emerged in the present study are emphasized. In order to meet this challenge, we suggest a co-design of research projects or implementation program to tailor the services in line with user needs. Co-design in healthcare is the act of designers in creating with stakeholders to ensure the results meet their needs and are usable [114]. This will hopefully, increase the use and usefulness of research in practice, and thus bridging the gap.

## Conclusions

Our findings delineate the means by which the gap between research-based knowledge and clinical practice that pertains to the implementation of the evidence-based OEP can be bridged. OEP was found to be a suitable framework that can guide clinical work and provide important research-based knowledge about fall prevention. The OEP unifies the goals of research and practice, enhances our understanding of physiotherapy, and serves as an adaptable knowledge base. The findings suggest that health workers and researchers need to acknowledge the importance of user-friendly language, the diversity in the sources of knowledge, and the tension that exists between these sources (i.e., contextual demands, research-based knowledge, clinical expertise, patient needs, values, and preferences)*.* The evidence-based OEP can be used to improve healthcare services and bridge the perceived and real gap between research and practice. Further research is needed. Based on the themes from the qualitative study, we suggest a survey with a bigger sample of patients who have completed OEP, or other fall prevention program. This will hopefully strengthen our findings, as well as expose more knowledge gaps in the implementation of fall prevention intervention in the municipality.

## Supplementary information

**Additional file 1.** Sample questions that were posed to the participants during the in-depth interview.

**Additional file 2.** A summary of the emergent subthemes, main themes, and overarching themes.

## Abbreviations

BBS: Berg Balance Scale
EBP: Evidence-based practice
MCS-12: Mental Component Summary (SF-12: The Short Form-12 test)
MMSE: Mini-Mental State Examination
OEP: Otago Exercise Programme
PCS-12: Physical Mental Component Summary (SF-12: The Short Form-12 test)
SPPB: Short Physical Performance Battery
