# Supplementary material for: Bridging the gap between research-based knowledge and clinical practice: a qualitative examination of patients and physiotherapists’ views on the Otago exercise Programme
Source: BMC Geriatr. 2019 Oct 21;19:278. doi: 10.1186/s12877-019-1309-6 (PMC6805671; doi:10.1186/s12877-019-1309-6)
Supplement: Supplementary file 1 — Additional file 1. Sample questions that were posed to the participants during the in-depth interview. [file 12877_2019_1309_MOESM1_ESM.docx]

**Sample questions that were posed to the participants during the in-depth interview**

| **Patients** | **Interview: 1 week after the completion of the Otago Exercise Programme**  Can you please describe your experience of participating in the exercise intervention?  Can you please describe if and how your needs and preferences were considered in the implementation of this exercise intervention?  Can you please describe what you consider to be the best aspect of the exercise program?  Can you please describe what you consider to be the worst aspect of the exercise program?  Can you please describe the nature of your relationship with the physiotherapist?  What is your opinion about the fact that this exercise program is based on research findings?  How do you think the exercise program can be improved? |
| --- | --- |
| **Physiotherapists** | **Interview: 1 week after the completion of the Otago Exercise Programme**  Can you please describe your experience of participating in the evidence-based Otago Exercise Programme?  Can you please describe your views about the research-based knowledge that pertains to fall prevention?  What are the factors that contribute to the successful implementation of a research-based program such as the Otago Exercise Programme? What do you think about the application of research-based knowledge in fall prevention interventions?  How can you and your college contribute to the application of research-based knowledge in fall prevention?  How can the application of research-based knowledge be improved? |
