# Supplementary material for: Bridging the gap between research-based knowledge and clinical practice: a qualitative examination of patients and physiotherapists’ views on the Otago exercise Programme
Source: BMC Geriatr. 2019 Oct 21;19:278. doi: 10.1186/s12877-019-1309-6 (PMC6805671; doi:10.1186/s12877-019-1309-6)
Supplement: Supplementary file 2 — Additional file 2. A summary of the emergent subthemes, main themes, and overarching themes. [file 12877_2019_1309_MOESM2_ESM.docx]

**A summary of the emergent subthemes, main themes, and overarching themes**

| **Examples of characteristic responses** | **Code** | **Subtheme** | **Main theme** | **Overarching theme** |
| --- | --- | --- | --- | --- |
| *An important point for success in implementation is academic humility. The researcher must not look at himself as infallible and super-intelligent ... they must show that they recognize others and respect others' views. They must not always be concerned about persuading.* | - Academic humility - Self-esteem - Respect - Communication style | *The researcher is full of himself* | The researcher’s role and position in the implementation process | A dynamic integrative process: successful implementation requires researchers, patients, clinicians, and organizations to modify established perceptions, behaviors, language, and practice into routine behaviors. |
| *I think it's nice that researchers become more interested in what the practice field believes and their experience base. I think that in itself will facilitate the implementation of research in practice. We would like to use something that we know works.* | - Clinical knowledge and interest - Sufficient knowledge about the field of practice. - Researchers’ interest in clinical practice | The "bottom-up" approach: collaborative interests |  |  |
| *We have to accept that we understand things differently and that somebody sometimes perceived that they do not fully understand what research is about. Ambiguity and acceptance of different degrees of understanding provide the opportunity for negotiation.* | - Understand things differently - Ambiguity and acceptance - Negotiation - Prioritization | The need for reflective or critical uncertainty in practice and research | The tension between research-based knowledge and clinical practice |  |
| *She sees you, she answers your questions in a simple way so I understand it ... she knows a lot and doesn't overrule you but talks about the most important things.*  *We know that it is not about filling up empty jars ... but have to be linked to the users' desires and preferences.* | - Not about *filling up empty jars* - Users’ desires - Users’ preferences | Creation of a mutual language and understanding across different users: translation leads to transformation |  |  |
|  |  | The patient’s lived experiences and the clinician’s knowledge |  |  |
